# Outcomes in Adenomyosis Treated with Uterine Artery Embolization Are Associated with Lesion Vascularity: A Long-Term Follow-Up Study of 252 Cases

**DOI:** 10.1371/journal.pone.0165610

**Published:** 2016-11-02

**Authors:** Jing Zhou, Li He, Ping Liu, Hui Duan, Hanze Zhang, Weili Li, Shipeng Gong, Guidong Su, Chunlin Chen

**Affiliations:** 1 Department of Obstetrics and Gynecology, Nanfang Hospital, Southern Medical University, Guangzhou, China; 2 Department of Ophthalmology, School of Medicine, Emory University, Atlanta, Georgia, United States of America; 3 Department of Epidemiology and Biostatistics, College of Public Health, University of South Florida, Tampa, Florida, United States of America; West China Second Hospital, Sichuan University, CHINA

## Abstract

**Purpose:**

To study the therapeutic effects of uterine artery embolization (UAE) on adenomyosis and to investigate the association between uterine blood supply and artery embolization treatment outcomes.

**Methods:**

Using digital subtraction angiography (DSA) imaging data, we retrospectively evaluated the vascular features of 252 adenomyosis patients treated with UAE. The cases were classified based on the equality of uterine blood supply (equal and unequal subgroups) and the degree of vascularity at the adenomyosis lesion site (hypervascular, isovascular and hypovascular subgroups). Patients were followed-up for 5 years after UAE. Improvements in dysmenorrhea and menorrhagia were evaluated based on the relief of the patients’ symptoms. The improvement rates among the different subgroups were analyzed and compared.

**Results:**

The improvement rates of dysmenorrhea and menorrhagia were 74.0% and 70.9%, respectively, at the short-term (12-month) follow-up and 70.4% and 68.8%, respectively, at the long-term (5-year) follow-up. No statistically significant differences were observed in the improvement rates for dysmenorrhea or menorrhagia between the equal and unequal blood supply subgroups at either the short- or long-term follow-up. The improvement rates for dysmenorrhea among the hypervascular, isovascular and hypovascular subgroups were 86.5%, 71.8% and 58.8%, respectively, at the short-term follow-up (*p = 0*.*002*) and 83.6%, 67.3% and 52.8%, respectively, at the long-term follow-up (*p = 0*.*005*). The improvement rates for menorrhagia in the hypervascular, isovascular and hypovascular subgroups were 81.0%, 68.3% and 60.7%, respectively, at the short-term follow-up (*p = 0*.*024*) and 79.4%, 61.4% and 62.2%, respectively, at the long-term follow-up (*p = 0*.*052*).

**Conclusion:**

UAE is effective in treating patients with adenomyosis in both the short and long term. The outcomes of patients with adenomyosis were significantly correlated with lesion vascularity.

## Introduction

Adenomyosis is a common gynecological condition characterized by the diffuse or local growth of endometrial glandular tissue, which invades the muscular layer of the uterus. This condition is primarily seen in women between 30 and 50 years of age, and it is usually the side-effect of an intrauterine procedure. The main symptoms of adenomyosis include dysmenorrhea, menorrhagia, infertility, and an enlarged uterus. The reported prevalence of adenomyosis varies from 5% to 70% because of differences in ethnicity, case selection, and diagnostic criteria [1]. To meet the individualized needs of patients, multiple treatment approaches have been widely used, including surgery, conservative treatment, hormone therapy, and interventional radiology or minimally invasive therapy [2, 3].

Uterine artery embolization (UAE) is a new treatment approach that was developed earlier this century. Since 2001, when Siskin reported using UAE to treat 15 patients with adenomyosis [4], it has been tested by different institutions and determined to be an effective treatment for adenomyosis, particularly in patients who are resistant to routine medicine therapy and those who wish to preserve their uterus [5–17]. In this vascular imaging-guided procedure, a gynecologist or interventional radiologist uses a catheter to deliver small particles to block the blood supply to targeted lesions, which are selected based on the diameter of the uterine artery and the degree of blood supply to the lesion. This procedure is advantageous because it is minimally invasive and maintains the patient’s fertility. In theory, UAE blocks the blood supply to both the uterus and the lesion, causing ischemic and hypoxic damage to the ectopic endometrium. The proliferated endometrial glandular cells and connective tissues will undergo necrosis and be scavenged, while the normal uterine tissue will atrophy but survive through collateral circulation. Most patients report satisfactory relief of dysmenorrhea and menorrhagia, although recurrence occurs in some cases based on long-term observations [18]. Therefore, it is necessary to validate the efficacy of UAE in larger patient populations and with longer follow-up periods and to identify the factors that can predict the therapeutic effects and facilitate decision-making for both trained gynecologists and patients.

In this study, we retrospectively analyzed data from 252 patients who underwent UAE, and we aimed to identify the vascular features of adenomyosis correlated with short- and long-term UAE outcomes.

## Materials and Methods

### Study design and population

The study design was retrospective, observational, and single-institute. From June 1999 to August 2008, 264 adenomyosis patients (mean age, 36.7 years; median age, 38 years; range, 28–51 years) who fulfilled these criteria were included in the study: a) diagnosed with adenomyosis by history, dysmenorrhea and/or menorrhagia symptoms, clinical examinations, and magnetic resonance imaging (MRI) results; b) exhibited resistance to continuous medicine therapy and expressed the desire to preserve the uterus. Patients with leiomyoma or other diseases that lead to anemia were excluded from this study. All patients were fully aware of the benefits and adverse effects of UAE and other treatment options. This study was approved by the Ethics Committee of Southern Medical University, and written informed consent was obtained from all patients prior to UAE.

### UAE procedure

UAE was performed under the guidance of digital subtraction angiography (DSA). The right femoral artery was used as an access route in all cases. A 5.0-F RHR catheter (Cook, Bloomington, IN, USA) was placed in the right internal iliac artery, and a coaxial 3-F microcatheter (MicroFerret; Cook, Bloomington, IN, USA) was advanced into the distal uterine artery. After placing the catheter tip beyond the origin of the right cervicovaginal branch, nonionic contrast media agent (Ultravist 370 mg Iodine /mL, Bayer Healthcare Pharmaceuticals, Berlin, Germany) was injected. Then, the microcatheter was advanced to the origin of the left cervicovaginal branch via common iliac and left internal iliac artery, and contrast media agent was injected. After obtaining both left and right uterine DSA image for vascularization analysis, bilateral embolization procedure was performed. In all cases, the primary embolic agent was 355-500-μm, 500-710-μm polyvinyl alcohol (PVA) particles (Contour, Boston Scientific, Marlborough, MA, USA) mixed with 40 mL of a 1:1 saline/contrast agent mixture to capture the angiography imaging data. This was followed by a secondary supplemental embolization with gelatin sponge pledgets. Embolization was performed until there was complete cessation of the blood flow in the ascending uterine artery and residual flow in the lower uterine segment. An imaging CD, which recorded the entire angiography and embolization procedure, was created for each case.

### Blood supply analysis and classification

We retrospectively reviewed the DSA images from each patient and developed two classification methods based on the blood supply equality of the uterus and vascularity degree of adenomyosis lesion. The blood supply equality and vascularity degree was determined by three experienced radiologists analyzing the DSA image together.

**Fig 1:**
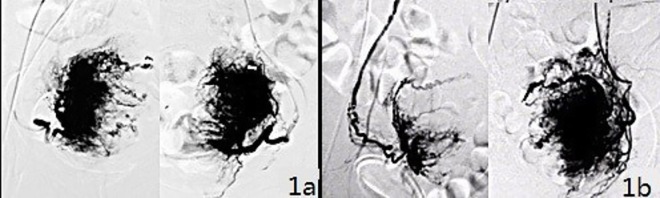
Classification of the distribution equality of the uterus blood supply. 1a) Similar blood supply levels from the left and right uterine arteries, which is defined as an equal blood supply. 1b) Significantly different blood supply levels from the left and right uterine arteries, which is defined as an unequal blood supply.

**Fig 2:**
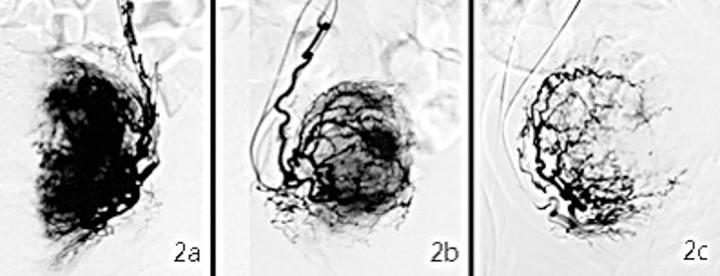
Classification of vascularity abundance for adenomyosis. 2a) An example of a hypervascular adenomyosis lesion, which shows a strong imaging signal; 2b) an Isovascular lesion with a moderate signal; and 2c) a hypovascular lesion with a weak signal.

1) Two subgroups were defined based on the distribution equality of the uterine blood supply. The equal subgroup had similar levels of blood supply from the left and right uterine arteries, meaning that each artery supplied approximately 40% to 60% of the total uterus (Fig 1A). The unequal subgroup had one uterine artery supplying more than 60% of the total uterus (Fig 1B).

2) Three subgroups were defined based on the degree of vascularity abundance. In the hypervascular subgroup, the vessels were abundant at both the margin and center of the lesions, and there were strong signals in the whole lesion (Fig 2A). In the isovascular subgroup, the vessels were rich in the peripheral areas but less so in the core of the lesions, which exhibited a moderate staining signal (Fig 2B). Finally, in the hypovascular subgroup, there was a lack of vessels at either the margin or the center of the lesions and a mild staining signal (Fig 2C).

### Follow-up and symptom grading

Each patient was interviewed by experienced gynecologist before undergoing UAE. Follow-up interviews were conducted in outpatient clinics or by telephone at 3 months, 6 months, 9 months, 12 months, 2 years, 3 years, 4 years, and 5 years after UAE.

A chronic pain grading questionnaire [19] was used to evaluate patients’ dysmenorrhea symptoms. The questionnaire graded pain using a score of 0 to 100, and activity loss was scored from 0 to IV. Grade 0 was defined as no pain and no loss of activity; grade I indicated a pain score < 50 and an activity loss score < 3; grade II indicated a pain score > 50 and an activity loss score < 3; grade III indicated an activity loss score of 3 or 4, regardless of pain score; and grade IV indicated an activity loss score of 5 or 6, regardless of pain score (Table 1).

**Table 1:** Dysmenorrhea symptom grades.

	Pain score	Activity loss score
Grade 0	0	0
Grade I	<50	<3
Grade II	>50	<3
Grade III	any	3 or 4
Grade IV	any	5 or 6

A grade of II, III or IV before UAE was defined as dysmenorrhea, while a grade of 0 or I indicated no dysmenorrhea symptoms. Following UAE, a reduction of 2 or more grades was defined as improvement in dysmenorrhea symptoms.

By analyzing the number of pads used during a menstrual period and the blood hemoglobin (HGB) level, menorrhagia symptoms were categorized as grade 0 to III. Grade 0 indicated amenorrhea; grade I indicated fewer than 20 pads used during a menstrual period and an HGB level > 110 g/L; grade II indicated fewer than 20 pads used and an HGB level < 110 g/L; and grade III indicated more than 20 pads used and an HGB level < 110 g/L. Grades II and III were defined as menorrhagia. A reduction of 1 or more grades after UAE was defined as improvement in menorrhagia symptoms (Table 2).

**Table 2:** Menorrhagia symptom grades.

	HGB g/L	Pads needed during a menstrual period
Grade 0	amenorrhea	
Grade I	>110	<20
Grade II	>110	>20
Grade III	<110	>20

Recurrence was defined when dysmenorrhea symptoms increased by 2 grades, when menorrhagia symptoms increased by 1 grade, or when symptoms reappeared according to a patient’s personal judgment. Patients who underwent a hysterectomy due to ineffectiveness or recurrence were considered to have completed the follow-up.

The short-term follow-up data included patients who were followed-up for 12 months. The long-term follow-up data included patients who were followed-up for 5 years. Lost to follow-up included refusal, the inability to make contact, death, or hysterectomy for other reasons. Patients who experienced ovarian failure in the 3 months following UAE and those with failed embolization procedures were excluded from the study.

### Statistical analysis

SPSS 15.0 software was used for the statistical analyses. The chi-square test was used to compare improvements in symptoms after UAE among the different subgroups. The *t*-test was used to compare the degree of symptoms in the different subgroups before UAE. Differences were considered to be statistically significant at *p*<0.05.

## Results

### Overall clinical outcomes

Of the 264 adenomyosis patients with symptoms of dysmenorrhea and/or menorrhagia who underwent UAE, 12 were excluded. Four had a failed embolization treatment, 7 were diagnosed with ovarian failure within three months after UAE, and 1 died of a pulmonary embolism the day after UAE. The remaining 252 patients (100%) completed the 12-month follow-up, and 195 (77.4%) patients completed the 5-year follow-up. Of the 57 patients lost to follow-up, 45 refused to continue or contact was lost; 9 had hysterectomies due to other diseases (2 cervical cancers, 5 cervical intraepithelial neoplasia III, and 2 postpartum hemorrhage); and 3 died from other causes.

Among the 252 patients who completed the 12-month follow-up, 145 of the 196 patients with dysmenorrhea (74.0%) reported improvement after UAE; 161 of the 227 patients with menorrhagia (70.9%) reported improvement after UAE; and 108 of the 252 patients (42.9%) experienced the recurrence of at least one symptom during follow-up.

Among the 195 patients who completed the 5-year follow-up, 107 of the 152 patients with dysmenorrhea (70.4%) reported improvement after UAE; 117 of the 170 patients with menorrhagia (68.8%) reported improvement after UAE; and 92 of the 195 patients (47.2%) experienced the recurrence of at least one symptom during follow-up.

### Improvement of dysmenorrhea

#### Blood supply equality subgroups

**Fig 3:**
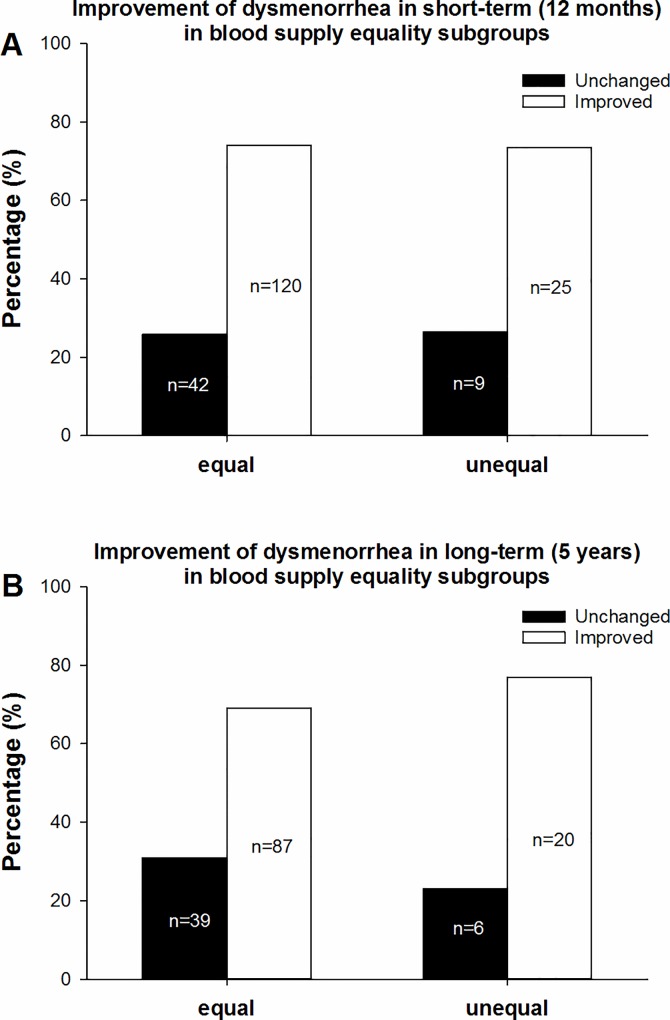
Improvement of dysmenorrhea in the blood supply subgroups. Fig 3a shows the percentages of improvement in dysmenorrhea at the short-term follow-up, including 74.1% (120/162) in the equal subgroup and 73.5% (25/34) in the unequal subgroup. Fig 3b shows the percentages of improvement in dysmenorrhea at the long-term follow-up, including 69.0% (87/126) in the equal subgroup and 76.9% (20/26) in the unequal subgroup. No statistically significant difference was observed between the equal and unequal subgroups at the short- or long-term follow-up.

According to our subgroup classification based on blood supply quality, the 196 patients with dysmenorrhea symptoms who completed the short-term follow-up were divided into two subgroups, with 162 (82.7%) in the equal subgroup and 34 (17.3%) in the unequal subgroup. There was no significant difference between the two subgroups in the grade of dysmenorrhea before UAE (*p = 0*.*992*). The percentages of improved cases in the equal and unequal subgroups were 74.1% (120/162) and 73.5% (25/34), respectively (Fig 3A). No significant difference in the short-term improvement of dysmenorrhea was observed between the two subgroups (*χ2 = 0*.*004*, *p = 0*.*948*) (Table 3).

**Table 3:** Improvement of dysmenorrhea based on blood supply equality.

time	equality of blood supply	improvement of dysmenorrhea			total	χ2	*P*
		unchanged	improved	percentage of improvement			
Short term	equal	42	120	74.1%	162	0.004	0.948
	unequal	9	25	73.5%	34		
	total	51	145	74.0%	196		
Long term	equal	39	87	69.0%	126	0.641	0.423
	unequal	6	20	76.9%	26		
	total	45	107	70.4%	152		

A total of 152 patients completed the long-term follow-up, including 126 (82.9%) in the equal subgroup and 26 (17.1%) in the unequal subgroup. There was no significant difference between the two subgroups in the grade of dysmenorrhea before UAE (*p = 0*.*917*). The percentages of improved cases in the equal and unequal subgroups were 69.0% (87/126) and 76.9% (20/26), respectively (Fig 3B). There was no significant difference between the two subgroups in the long-term improvement of dysmenorrhea (*χ2 = 0*.*641*, *p = 0*.*423*) (Table 3).

#### Vascularity subgroups

**Fig 4:**
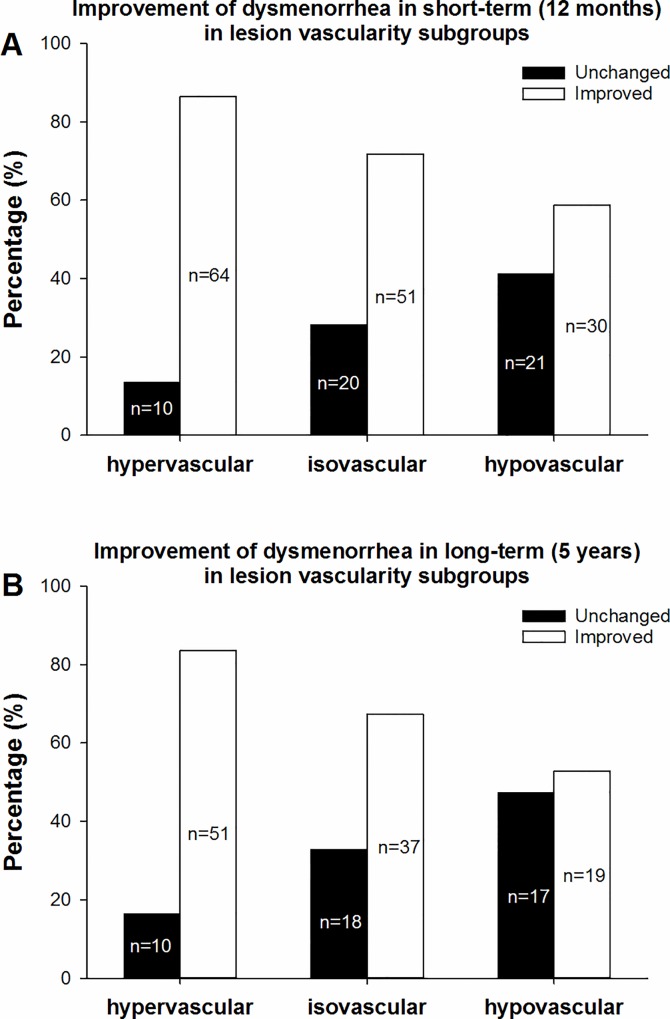
Improvement in dysmenorrhea in the different vascularity subgroups. In Fig 4a, which shows the short-term treatment effect, the percentage of improved cases in the hypervascular subgroup was the highest (86.5%, 64/74), followed by the isovascular subgroup (71.8%, 51/71) and the hypovascular subgroup (58.8%, 30/51), and these differences were statistically significant (χ2 = 12.269, p = 0.002). In Fig 4b, which shows the long-term effect, the percentages of improved cases in the hypervascular, isovascular, and hypovascular subgroups were 83.6% (51/61), 67.3% (37/55) and 52.8% (19/36), respectively. The differences among the three subgroups were statistically significant (χ2 = 10.727, p = 0.005).

According to our subgroup classification based on vascularity, the 196 patients with symptoms of dysmenorrhea who completed the short-term follow-up were divided into three subgroups, including 74 (37.8%) in the hypervascular subgroup, 71 (36.2%) in the isovascular subgroup, and 51 (26.0%) in the hypovascular subgroup. There was no statistically significant difference in the grade of dysmenorrhea before UAE among the three subgroups (*p = 0*.*905*). The highest percentage of improved cases was in the hypervascular subgroup (86.5%, 64/74), followed by the isovascular subgroup (71.8%, 51/71) and the hypovascular subgroup (58.8%, 30/51). (Fig 4A) The differences among the three subgroups were statistically significant (*χ2 = 12*.*269*, *p = 0*.*002*) (Table 4).

**Table 4:** Improvement of dysmenorrhea based on vascularity.

time	vascularity	improvement of dysmenorrhea			total	χ2	*P*
		unchanged	improved	percentage of improvement			
Short term	hypervascular	10	64	86.5%	74	12.269	0.002
	isovascular	20	51	71.8%	71		
	hypovascular	21	30	58.8%	51		
	total	51	145	74.0%	196		
Long term	hypervascular	10	51	83.6%	61	10.727	0.005
	isovascular	18	37	67.3%	55		
	hypovascular	17	19	52.8%	36		
	total	45	107	70.4%	152		

Among the 152 patients who completed the long-term follow-up, 61 (40.1%) were hypervascular, 55 (36.2%) were isovascular, and 36 (23.7%) were hypovascular. There was no statistically significant difference in the grade of dysmenorrhea before UAE among the three subgroups (*p = 0*.*817*). The percentages of improved cases in the hypervascular, isovascular, and hypovascular subgroups were 83.6% (51/61), 67.3% (37/55) and 52.8% (19/36), respectively, (Fig 4B). The differences among the three subgroups were also statistically significant (*χ2 = 10*.*727*, *p = 0*.*005*) (Table 4).

### Improvement of menorrhagia

#### Blood supply equality subgroups

**Fig 5:**
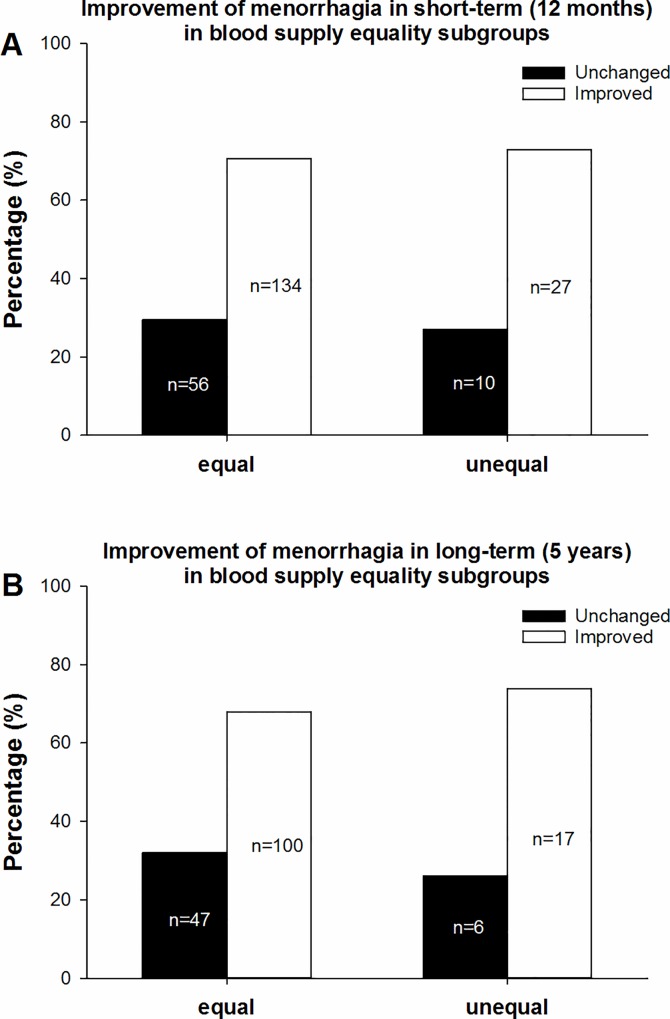
Improvement of menorrhagia in the blood supply subgroups. Fig 5a shows the percentages of improvement in menorrhagia at the short-term follow-up, including 70.5% (134/190) in the equal subgroup and 73.0% (27/37) in the unequal subgroup. Fig 5b shows the percentages of improvement in menorrhagia at the long-term follow-up, including 68.0% (100/147) in the equal subgroup and 73.9% (17/23) in the unequal subgroup. No statistically significant differences were observed between the equal and unequal subgroups at either the short- or long-term follow-up.

According to our subgroup classification based on blood supply equality, the 227 patients with menorrhagia symptoms who completed the short-term follow-up were divided into two subgroups, including 190 patients (83.7%) in the equal subgroup and 37 patients (16.3%) in the unequal subgroup. There was no significant difference between the two subgroups in the grade of menorrhagia before UAE (*p = 0*.*268*). The percentages of improved cases in the equal and unequal subgroups were 70.5% (134/190) and 73.0% (27/37), respectively (Fig 5A). There was no significant difference between the two subgroups (*χ2 = 0*.*09*, *p = 0*.*764*) (Table 5).

**Table 5:** Improvement of menorrhagia based on blood supply equality.

time	equality of blood supply	improvement of menorrhagia			total	χ2	*P*
		unchanged	improved	percentage of improvement			
Short term	equal	56	134	70.5%	190	0.09	0.764
	unequal	10	27	73.0%	37		
	total	66	161	70.9%	227		
Long term	equal	47	100	68.0%	147	0.321	0.571
	unequal	6	17	73.9%	23		
	total	53	117	68.8%	170		

A total of 170 patients completed the long-term follow-up, including 147 (86.5%) in the equal subgroup and 23 (13.5%) in the unequal subgroup. There was no significant difference between the two subgroups in the grade of dysmenorrhea before UAE (*p = 0*.*195*). The percentages of improved cases in the equal and unequal subgroups were 68.0% (100/147) and 73.9% (17/23), respectively (Fig 5B). There was no significant difference between the two subgroups (*χ2 = 0*.*321*, *p = 0*.*571*) (Table 5).

#### Vascularity subgroups

**Fig 6:**
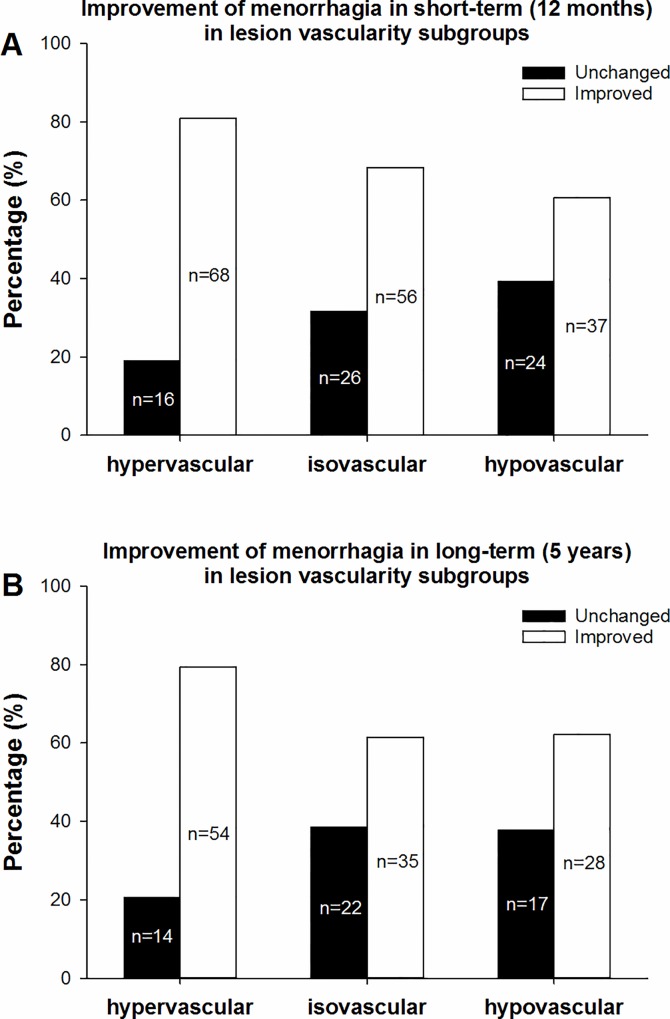
Improvement of menorrhagia in the vascularity subgroups. In Fig 6a, the hypervascular subgroup had the highest percentage of short-term improvement in menorrhagia (81.0%, 68/84), followed by the isovascular subgroup (68.3%, 56/82) and the hypovascular subgroup (60.7%, 37/61). A statistically significant difference was observed among the three subgroups (χ2 = 7.491, p = 0.024). In Fig 6b, which shows the long-term effect, the percentages of improved cases in the hypervascular, isovascular, and hypovascular subgroups were 79.4% (54/68), 61.4% (35/57) and 62.2% (28/45), respectively. There was no statistically significant difference among the three subgroups (χ2 = 5.929, p = 0.052).

According to our subgroup classification based on vascularity, the 227 patients with menorrhagia symptoms who completed the short-term follow-up were divided into three subgroups, with 84 (37%) in the hypervascular subgroup, 82 (36.1%) in the isovascular subgroup, and 61 (26.9%) in the hypovascular subgroup. There was no significant difference in the grade of menorrhagia before UAE among the three subgroups (*p = 0*.*076*). The percentages of improved cases in the hypervascular, isovascular, and hypovascular subgroups were 81.0% (68/84), 68.3% (56/82) and 60.7% (37/61), respectively (Fig 6A), and the differences among the three subgroups were statistically significant (*χ2 = 7*.*491*, *p = 0*.*024*) (Table 6).

**Table 6:** Improvement of menorrhagia based on vascularity.

time	vascularity	improvement of menorrhagia			total	χ2	*P*
		unchanged	improved	percentage of improvement			
Short term	hypervascular	16	68	81.0%	84	7.491	0.024
	isovascular	26	56	68.3%	82		
	hypovascular	24	37	60.7%	61		
	total	66	161	70.9%	227		
Long term	hypervascular	14	54	79.4%	68	5.929	0.052
	isovascular	22	35	61.4%	57		
	hypovascular	17	28	62.2%	45		
	total	53	117	68.8%	170		

Among the 170 patients who completed the long-term follow up, 68 (40.0%) were in the hypervascular subgroup, 57 (33.5%) were in the isovascular subgroup, and 45 (26.5%) were in the hypovascular subgroup. There was no significant difference in the grade of menorrhagia before UAE among the three subgroups (*p = 0*.*179*). The percentages of improved cases in the hypervascular, isovascular, and hypovascular subgroups were 79.4% (54/68), 61.4% (35/57) and 62.2% (28/45), respectively (Fig 6B). There was no statistically significant difference among the three subgroups (*χ2 = 5*.*929*, *p = 0*.*052*) (Table 6).

## Discussion

The results from this study suggested that UAE is an effective short- and long-term treatment for women with therapy-resistant adenomyosis. Two major symptoms presented by most patients in this study, dysmenorrhea and menorrhagia, were significantly relieved. The short-term (74.0% for dysmenorrhea and 70.9% for menorrhagia) and long-term (70.4% for dysmenorrhea and 68.8% for menorrhagia) improvement rates demonstrated in this study are consistent with the review by *Popovic et al*. [18], who summarized the literature available before 2011 and reported a short-term improvement rate of 83.8% after an average of 9.4 months (102 cases) and a long-term improvement rate of 64.9% after an average of 40.6 months (208 cases). In 2012, *Smeets et al*. [13] studied 40 women and reported similar findings, with 29 of the 40 cases (72.5%) showing improvement. Our current data strongly add to the evidence of UAE as an efficient method of treating adenomyosis, particularly in patients who are resistant to conventional therapy and those wish to preserve their uterus.

More importantly, our results indicated that the degree of vascularity in adenomyosis lesions is associated with improvement of symptoms after UAE, meaning that better-vascularized lesions show a better response to UAE (Figs 4 and 6). Although the chi-square test showed a value of *p = 0*.*052* for the long-term improvement of menorrhagia in the different vascularity subgroups, there was a similar trend with the short-term improvement. However, we did not observe a significant relationship between the blood supply equality of uterus and the therapeutic effect (Figs 3 and 5). Therefore, we conclude that the vascularity of an adenomyosis lesion may be a useful predictor for the therapeutic effect of UAE. Regarding other potential predictors of UAE treatment outcomes, *Jung et al*. [11] studied 119 MR images from patients with adenomyosis before UAE and suggested that the T2-weighted signal intensity ratio was significantly associated with complete necrosis after UAE. Based on an analysis of 40 patients, *Kim et al*. [9] suggested that a dark MRI signal intensity (SI) of adenomyosis is the most favorable predictive factor for UAE, followed by a low SI, while a heterogeneous SI or an SI equal to that of the myometrium is an unfavorable predictive factor. Based on these findings, the pre-operational MRI signal can be a useful predictor of necrosis in adenomyosis lesions. Our data, which proved that the vascularity of adenomyosis lesions detected through angiography is a reliable outcome predictor, directly reinforce the previous findings. Interestingly, studies have suggested that the presence or absence of fibroids in addition to adenomyosis did not affect the clinical outcomes of UAE [7, 10, 17].

Although UAE showed a satisfactory therapeutic effect against adenomyosis in the short and long term, recurrence is an important prognostic factor that researchers cannot neglect. In our study, the recurrence rates were 42.9% (108 out of 252 patients) at the short-term follow-up and 47.2% (92 out of 195 patients) at the long-term follow-up. Based on two studies of relatively large population, *Kim et al*. [7] first reported a recurrence rate of 35.2% (19 of 54 patients) after UAE with a mean interval of 17.3 months. *Bae*, *et al*. [15] reported a 24% recurrence rate (12 of 50 patients) within 12 to 48 months, and the authors suggested that the percentage of lesion necrosis can be a predictor of symptom recurrence. There are several factors which may lead to the relatively high recurrence rate in our study: 1) most of patients included in this study were previously treated with other therapeutic approaches but exhibited resistance. Thus, these patients may represent a “refractory” adenomyosis. 2) Around 60% of included patients were categorized as isovascular or hypovascular adenomyosis lesion, which are relatively insensitive to UAE treatment. Therefore, it is desirable to pre-determine the vascularity of adenomyosis lesion before UAE procedure, meanwhile improve the UAE approach to achieve a higher success rate and a lower recurrence rate.

To the best of our knowledge, this report is the first to provide a vascularity analysis of adenomyosis treated with UAE. However, the classification of lesion vascularity was based on DSA imaging obtained during UAE procedures, which limits the value of prediction before treatment. Thus, we have been developing a three-dimensional reconstruction of uterine vessels with data from enhanced computerized tomographic angiography. An in vivo female pelvic arterial network model could be constructed with data collected through computerized tomographic angiography. This method could potentially be used to evaluate the abundance of vascularity before UAE, to predict outcomes and monitor probability of re-opening of embolized artery after UAE.

## Conclusions

Our study of 252 patients with adenomyosis treated with UAE confirmed the good short- and long-term clinical outcomes reported in previous studies. We are the first to report that lesion vascularity in adenomyosis predicts UAE outcomes, which means that better-vascularized lesions respond better to UAE treatment.
